# Long-term ozone exposure and the risk of mortality in sepsis patients in a Chinese cohort

**DOI:** 10.3389/fpubh.2026.1782393

**Published:** 2026-06-19

**Authors:** Weiwei Xiao, Yanqi Zhu, Fang Chen, Yan Cao, Yu Jiang, Yimin Zhu

**Affiliations:** 1 Basic Medical College, Health Science Center, Hunan Normal University, Changsha, Hunan, China; 2 Medical Science and Technology Development Center, Hunan Provincial Health Commission, Changsha, Hunan, China; 3 Hunan Provincial Key Laboratory of Emergency and Critical Care Metabonomics, Institute of Emergency Medicine, Hunan Provincial People’s Hospital (The First Affiliated Hospital of Hunan Normal University), Changsha, Hunan, China; 4 Department of Emergency Medicine, Hunan Provincial People’s Hospital (The First Affiliated Hospital of Hunan Normal University), Changsha, Hunan, China; 5 Geriatric Sepsis Research Center of Hunan Provincial Geriatric Institute, Hunan Provincial People’s Hospital (The First Affiliated Hospital of Hunan Normal University), Changsha, Hunan, China; 6 Health Science Center, Hunan Normal University, Changsha, Hunan, China

**Keywords:** environmental pollution, long-term exposure, mortality, ozone, sepsis

## Abstract

**Background:**

Air pollution is increasingly recognized as an important environmental risk factor for sepsis patients. However, previous studies have mostly focused on particulate matter, and few have explored the mortality risk associated with long-term ozone exposure among patients with sepsis, especially in China.

**Materials and methods:**

A cohort of patients with sepsis was recruited from 13 prefecture-level cities in Hunan Province, China. Annual mean ozone concentrations were obtained from a high-quality, high-resolution (1 × 1 km^2^) dataset of ground-level air pollutants in China. Logistic regression models were constructed to estimate the association between long-term ozone exposure and mortality risk among sepsis patients. Restricted cubic spline models were used to evaluate the dose–response relationship. Potential effect modification by age and sex was also examined through stratified analysis. To ensure the robustness of the results, we also performed a series of sensitivity analyses.

**Results:**

The study included 4,231 sepsis patients, among whom 151 deaths were recorded. We found that long-term ozone exposure significantly increased the risk of mortality in sepsis patients, with an odds ratio (OR) of 1.087 (95% CI, 1.020–1.158). Even after adjusting for PM_1_, PM_2.5_, and PM_10_, the association remained significant. Restricted cubic spline analysis revealed a monotonic increasing trend at lower ozone concentrations, with the risk of mortality becoming apparent when ozone levels exceeded 89.65 μg/m^3^. In the stratified analysis, the association between long-term ozone exposure and mortality risk was more pronounced among female individuals and children under 12 years of age, with *p*-for interaction <0.05.

**Conclusion:**

Long-term ozone exposure is associated with an elevated risk of mortality among sepsis patients, particularly among female individuals and children. Our study suggests that ozone is a potentially modifiable risk factor for mortality in this population, highlighting the need for improved air quality management and enhanced protection of vulnerable groups.

## Introduction

Sepsis is a life-threatening condition characterized by organ dysfunction resulting from a dysregulated host response to infection (1). It can rapidly lead to organ damage, shock, and death, contributing significantly to the global disease burden. Despite a decline in the age-standardized incidence and mortality rates of sepsis in recent years, it remains one of the leading causes of health loss worldwide (2). This disease burden is also present in China (3). A systematic review of the epidemiology of sepsis in mainland China reported that the average 30-day mortality rate among patients with sepsis is 29.5%, which is higher than that observed in other regions (4). Given this substantial disease burden, it is essential to identify the risk factors associated with both the onset of sepsis and sepsis-related mortality.

Air pollution, a ubiquitous environmental exposure, can increase the risk of chronic lung diseases, cardiovascular and cerebrovascular disorders, and other chronic diseases (5–7), which, in turn, can escalate the risk of sepsis (8, 9). Previous observational studies have also confirmed that air pollution is associated with increased morbidity and mortality in sepsis; however, these studies have mainly focused on PM_2.5_ (10–13). Only a few studies have examined the association between ozone exposure and sepsis. To the best of our knowledge, only two studies in mainland China have explored the effects of short-term ozone exposure on the incidence of sepsis (14, 15), and research on the prognosis of sepsis patients remains limited.

Ozone is of particular concern due to its potent oxidative capacity (16). Long-term, but not short-term, ozone exposure is significantly associated with an increased risk of respiratory death (17). Ozone pollution exposure has been linked to increased levels of pulmonary oxidative stress and inflammation (18, 19), thereby predisposing individuals to sepsis triggered by common infections. Moreover, ozone pollutant inhalation can activate multiple inflammatory signaling pathways, thereby potentially leading to immune dysregulation in patients with sepsis (20). This provides a plausible explanation for the association between ozone pollution and sepsis-related mortality. Considering that mainland China is heavily affected by ozone pollution, the associated mortality risk warrants serious attention.

To address these issues in mainland China, we performed a population-based cohort study using hospitalization data to explore the impact of long-term exposure to high levels of ozone on mortality among sepsis patients. Meanwhile, we further explored differences in susceptibility to ozone exposure through stratified analyses by age and sex.

## Materials and methods

### Study population

The study cohort was derived from the hospital information system (HIS) of more than 100 medical institutions in 13 prefecture-level cities in Hunan Province, China. We included a total of 6,782 inpatients from medical institutions at all levels who were admitted with sepsis from 1 January 2019 to 31 December 2022. Patients with sepsis were diagnosed by an experienced clinician based on the ICD-10 codes. Individuals hospitalized for more than 1,000 days (*n* = 236) and those with missing environmental data due to the absence of a current address (*n* = 2,315) were excluded. Ultimately, 4,231 individuals were included in the analysis. A detailed flowchart is presented in Supplementary Figure S1.

### Exposure assessment

The ozone and particulate matter data were obtained from the ChinaHighAirPollutants (CHAP) dataset^1^, which is based on multi-source satellite remote sensing technology and a four-dimensional spatiotemporal extreme random tree model. It combines big data such as ground-based observations, atmospheric reanalysis, and emission inventories to produce global, seamless, daily ground-level pollutant data. The spatial resolution of ozone in this dataset is 1 × 1 km^2^, and it has the characteristics of a long time series, full coverage, and high precision (21).

The annual average concentration of air pollutants from the CHAP dataset was matched for each patient according to the current address recorded in the HIS. Using the year of admission as the index year, the annual average exposure concentration for each patient was calculated over the 0–5 years preceding the index year. For example, the 1-year prior exposure was calculated as the mean of the average exposure concentrations in the index year and the year before the index year, and so on (22).

### Outcome definition

The primary outcome of this study was in-hospital mortality among patients with sepsis, including all-cause mortality. The cutoff date for recording mortality data was 31 December 2022, which marked the end of the study period. Secondary outcomes included severe and complicated conditions experienced by patients during their hospital stay, specifically any acute, serious, or complex medical complications.

### Ascertainment of covariates

Demographic data, including age, sex, and current address, were recorded by a trained clinician based on patients’ responses. Comorbidities and complications included diabetes, hypertension, pneumonia, upper respiratory tract infection (URTI), intracranial infection, and others. These data were obtained from the HIS, and all diagnoses were made by clinicians according to uniform criteria. In addition, we also extracted patients’ surgical status and length of hospital stay from the HIS.

### Statistical analysis

Baseline characteristics of the study participants were expressed as medians and IQRs for continuous variables, as all continuous variables were non-normally distributed, and as frequencies and percentages for categorical variables. Differences between the deceased and surviving populations were compared using the Wilcoxon test and the *χ*^2^ test, respectively.

A total of five logistic regression models were constructed to estimate the association between long-term exposure to ozone and the risk of mortality among patients with sepsis. In the crude model, adjustments were made for factors including age, sex, surgical status, and length of hospital stay. The adjusted model further controlled for comorbidities and complications, including diabetes, hypertension, pneumonia, upper respiratory tract infection (URTI), and intracranial infection. To further determine the robustness of the results, we additionally adjusted for other environmental contaminants. Two-pollutant models were constructed by separately adding PM_1_, PM_2.5_, and PM_10_ into the adjusted model. We used logistic regression analysis to assess the association between ozone exposure and the secondary outcomes using the adjusted model. In addition, given the role of the inflammatory response in the progression of sepsis, we used multiple linear regression analysis to evaluate the association between ozone exposure and C-reactive protein levels.

Restricted cubic spline analysis was also performed to demonstrate the dose–response relationship between ozone exposure and the risk of mortality among patients with sepsis. Furthermore, stratified analyses were conducted by age and sex. We conducted age-stratified analyses across four distinct age groups: 0–11 years (children), 12–18 years (adolescents), 19–65 years (adults), and ≥66 years (older adults). In the sensitivity analysis, we reanalyzed the data after separately excluding individuals with each of the following conditions: diabetes, hypertension, pneumonia, URTI, and intracranial infection.

The results were expressed as odds ratios (ORs) with 95% confidence intervals (95%CIs). All *p*-values were two-sided, and a value lower than 0.05 was considered statistically significant. All statistical analyses and graphical visualizations were performed using Stata version 17.0 or R version 4.4.1.

## Results

### Baseline characteristics of the study population

Table 1 presents the baseline characteristics of the participants according to survival status. The study included 4,231 sepsis patients, among whom 151 died. Overall, 72.3% of participants were younger than 18 years, and 56.72% were male. Compared to survivors, patients who died were more likely to be aged over 65 years, had longer hospital stays and higher C-reactive protein levels, and had a higher prevalence of diabetes and hypertension. The non-survivor group was generally exposed to higher ozone concentrations than the survivor group. Similar patterns were observed for the three types of particulate matter. Ozone exposure data for other years are provided in Supplementary Table S1.

**Table 1:** Baseline characteristics of the participants included in the study.

Numbers	Total population	Deaths	Survivors	*p*
	4,231	151	4,080	
Sex				
Male	2,400 (56.72)	27 (17.88)	2,373 (58.16)	0.954
Female	1718 (40.61)	19 (12.58)	1,699 (41.64)	
Unknown	113 (2.67)	105 (69.54)	8 (0.20)	
Age groups				
Children	2,998 (70.86)	17 (11.26)	2,981 (73.06)	<0.001
Adolescents	61 (1.44)	2 (1.32)	59 (1.45)	
Adults	497 (11.75)	34 (22.52)	463 (11.35)	
Older adults	573 (13.54)	97 (64.24)	476 (11.67)	
Unknown	102 (2.41)	1 (0.66)	101 (2.48)	
Surgery	371 (8.77)	7 (4.64)	364 (8.92)	0.067
Length of hospital stay (days)	6 (4, 8)	9 (5, 14)	6 (4, 8)	<0.001
C-reaction protein (mg/L)	27.62 (9.19, 72.15)	51.73 (31.35, 163.68)	27.26 (9.13, 71.90)	0.006
Comorbidity				
Pneumonia	653 (15.43)	16 (10.60)	637 (15.61)	0.094
URTI	1,277 (30.18)	6 (3.97)	1,271 (31.15)	<0.001
Diabetes	116 (2.74)	21 (13.91)	95 (2.33)	<0.001
Hypertension	141 (3.33)	32 (21.19)	109 (2.67)	<0.001
Intracranial infection	193 (4.56)	1 (0.66)	192 (4.71)	0.019
Severe and complicated conditions	127 (3.00)	2 (1.32)	125 (3.06)	0.327
Air pollutants				
O_3_ (μg/m^3^)	89.65 (87.20, 94.85)	95.55 (91.05, 96.65)	89.05 (87.20, 94.75)	<0.001
PM_1_ (μg/m^3^)	16.75 (15.70, 19.40)	22.05 (19.20, 22.05)	16.70 (15.70, 19.20)	<0.001
PM_2.5_ (μg/m^3^)	29.60 (27.60, 33.95)	39.20 (34.75, 39.35)	29.60 (27.60, 33.95)	<0.001
PM_10_ (μg/m^3^)	52.20 (45.60, 55.90)	56 (53.25, 56.10)	52.05 (45.60, 55.85)	<0.001

URTI, upper respiratory tract infection; PM_1_, particulate matter with aerodynamic diameter ≤1 μm; PM_2.5_, particulate matter with aerodynamic diameter ≤2.5 μm; PM_10_, particulate matter with aerodynamic diameter ≤10 μm; O_3_, ozone. Continuous variables were expressed as medians and IQRs, and categorical variables were expressed as frequencies and percentages.

### The association between ozone exposure and mortality in sepsis patients

**Figure 1:**
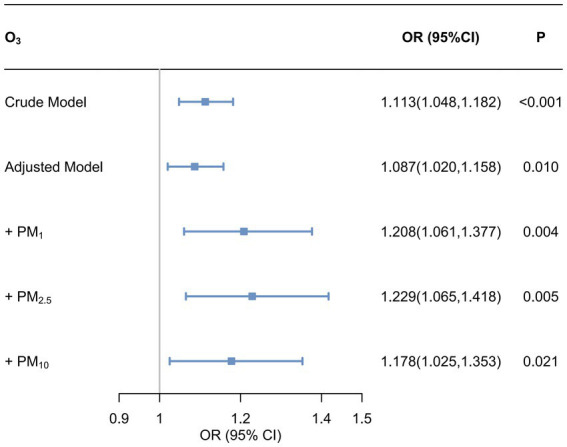
Association between ozone exposure and mortality in sepsis patients. Crude model: Adjusted for baseline age, sex, surgery, and length of hospital stay. Adjusted model: Further adjusted for pneumonia, upper respiratory tract infection (URTI), diabetes, hypertension, and intracranial infection based on the crude model. +PM_1_: Two-pollutant models, adding PM_1_ to the adjusted model; +PM_2.5_: Two-pollutant models, adding PM 2.5 to the adjusted model; +PM_10_: Two-pollutant models, adding PM_10_ to the adjusted model. PM_1_, particulate matter with aerodynamic diameter ≤1 μm; PM_2.5_, particulate matter with aerodynamic diameter ≤2.5 μm; PM_10_, particulate matter with aerodynamic diameter ≤10 μm; O_3_, ozone; OR, odds ratio; CI, confidence interval.

According to the results of the preliminary analysis, the preceding 1-year exposure period was selected as the primary exposure window in this study due to its strongest effect on mortality (Supplementary Figure S2). The OR and 95% CI for mortality in sepsis patients associated with ozone exposure under different models are presented in Figure 1. The results indicated that long-term ozone exposure significantly increased the risk of mortality among sepsis patients across all models. In the crude and adjusted models, the OR for mortality in sepsis patients was 1.113 (95% CI, 1.048, 1.182) and 1.087 (95%CI, 1.020, 1.158), respectively. Furthermore, we also found that long-term exposure to ozone was associated with an increased risk of severe and complicated conditions in patients with sepsis (Supplementary Figure S3). We observed that long-term exposure to ozone was positively correlated with the level of the inflammatory marker C-reactive protein (CRP) in patients with sepsis (Supplementary Table S2).

**Figure 2:**
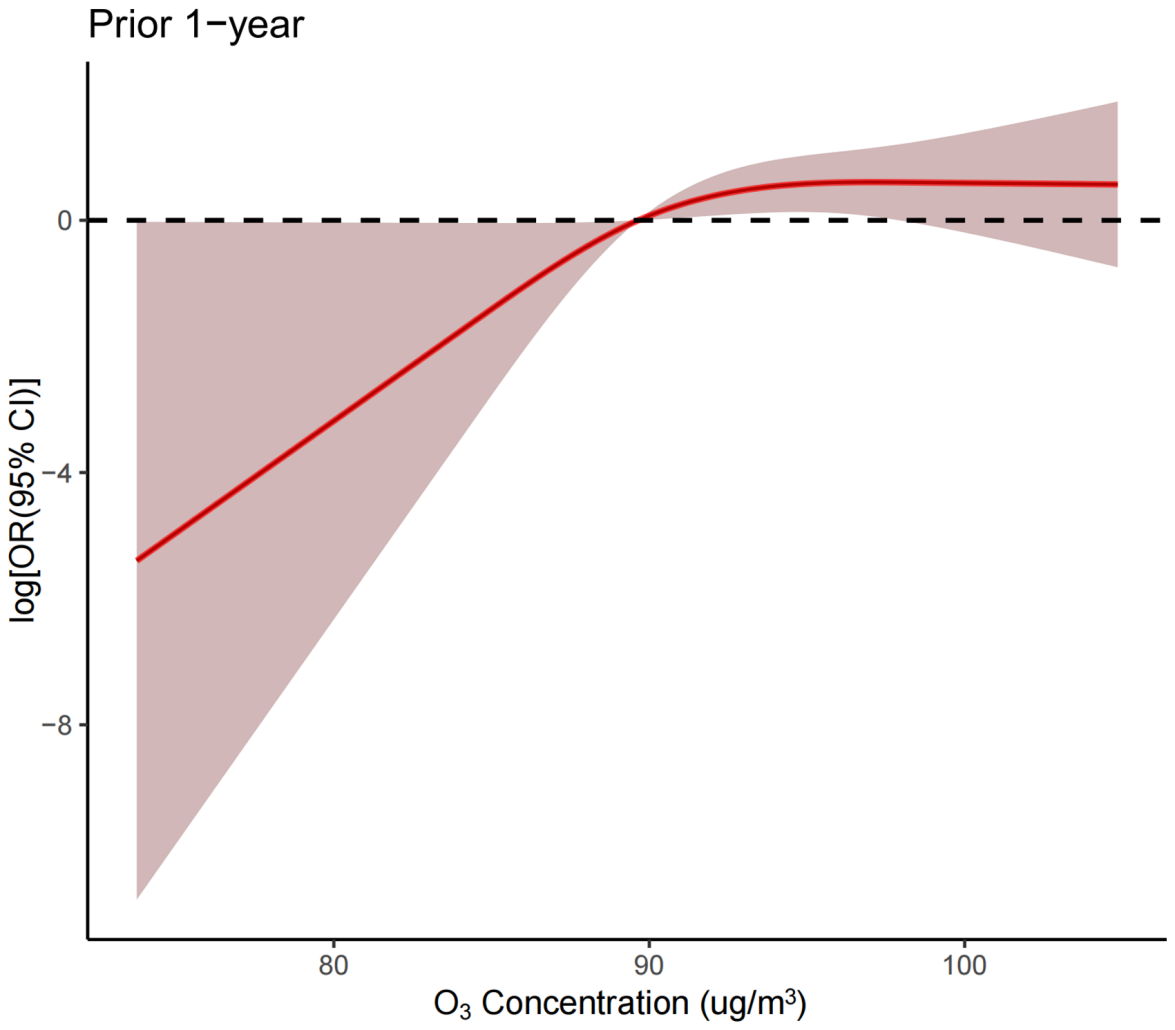
Exposure–response curves for the association between long-term ozone exposure and mortality risk in sepsis patients. Adjusted for baseline age, sex, surgery, length of hospital stay, pneumonia, upper respiratory tract infection (URTI), diabetes, hypertension, and intracranial infection. The shaded area represents 95% CIs. O_3_, ozone; OR, odds ratio; CI, confidence interval.

Given the effect of particulate matter on the risk of mortality in sepsis patients, we also investigated the association between particulate matter exposure and mortality in sepsis patients (Supplementary Table S3). An increased mortality risk was observed only for PM_10_ exposure, with an OR of 1.070 (95% CI, 1.010, 1.133). Figure 1 also presents the results of the two-pollutant models, which were used to account for potential confounding by PM. The effect estimates remained significant and were even strengthened after additional adjustment for PM_1_, PM_2.5_, and PM_10_. The ORs were 1.208 (95% CI, 1.061, 1.377), 1.229 (95% CI, 1.065, 1.418), and 1.178 (95% CI, 1.025, 1.353) in the two-pollutant models adjusted for PM_1_, PM_2.5_, and PM_10_, respectively. Restricted cubic spline analysis revealed a monotonic increasing trend at lower ozone concentrations, with the risk of mortality becoming apparent only when ozone levels exceeded 89.65 μg/m^3^. At higher concentrations, the dose–response curves reached a plateau or showed a slight decrease in risk (Figure 2).

### Sensitivity analysis and stratification analysis

**Figure 3:**
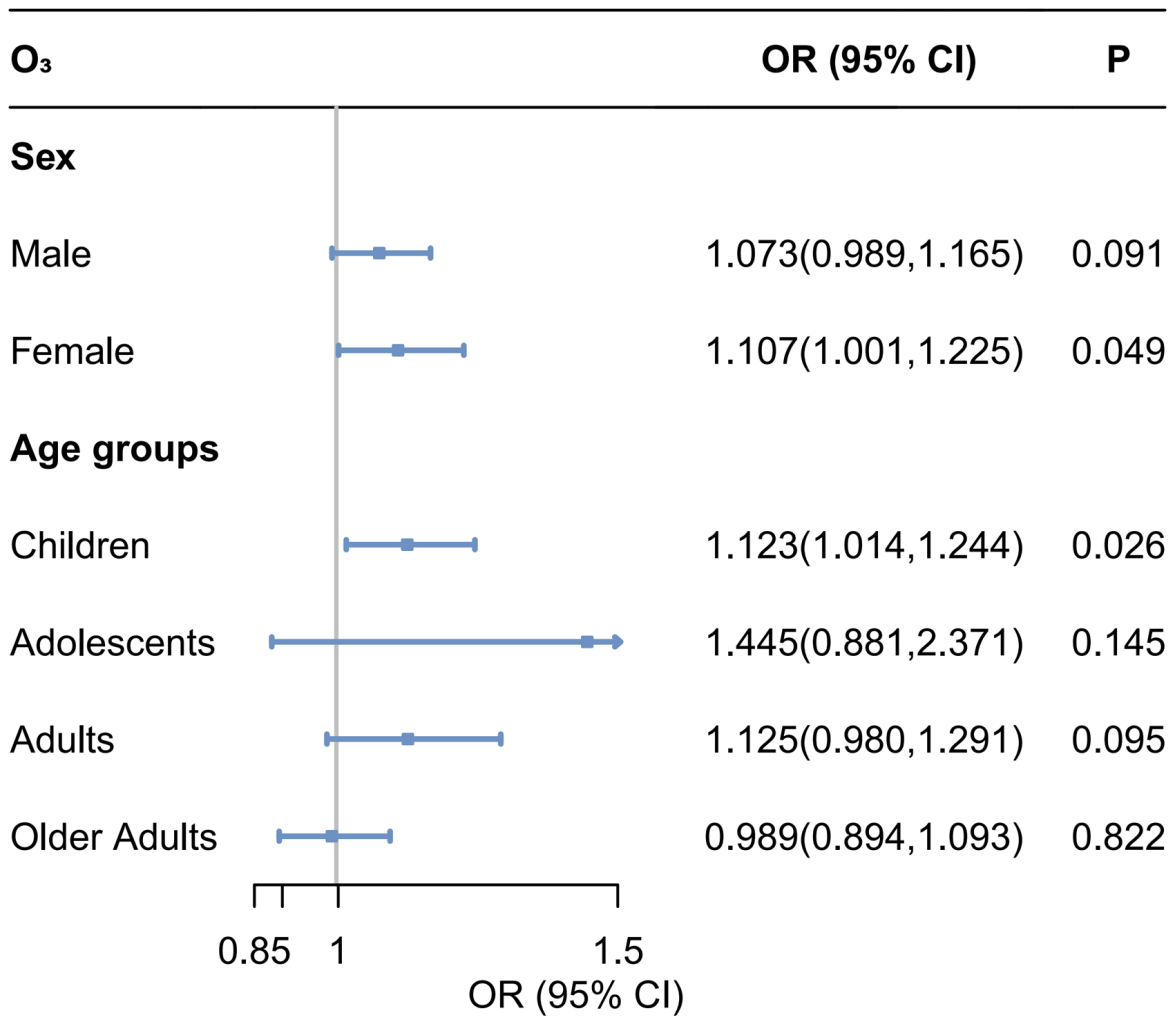
Associations between ozone exposure and the risk of mortality in sepsis patients, stratified by sex and age. An adjusted model was used. O_3_, ozone; OR, odds ratio; CI, confidence interval.

In the sensitivity analysis, the pattern of association between ozone exposure and the risk of mortality in sepsis patients did not change significantly when we separately excluded participants with diabetes, hypertension, pneumonia, upper respiratory tract infection (URTI), and intracranial infection (Supplementary Figure S4). In the sex-stratified analysis, the association between ozone exposure and the risk of mortality in sepsis patients remained significant in female individuals but not in male individuals (Figure 3). In the age-stratified analysis, the strongest association between ozone exposure and the risk of mortality in sepsis patients was observed in children younger than 12 years. The age-stratified results for C-reactive protein were also consistent (Supplementary Table S2). These results suggest that female individuals and children are more susceptible to the adverse effects of ozone exposure.

## Discussion

In this study, we investigated the association between long-term ozone exposure and mortality in sepsis patients across 13 cities in Hunan Province, China. We finally found that long-term ozone exposure increased the risk of mortality in sepsis patients, especially in female individuals and children. This association may be mediated through inflammatory processes. Restricted cubic spline analysis revealed that the association became more pronounced when ozone concentrations exceeded 89.65 μg/m^3^. The results of this study add to the evidence on the adverse health effects of long-term exposure to high levels of air pollution (23), suggesting that reducing ozone air pollution may yield benefits for sepsis patients.

Previous studies have mainly been conducted in European and American countries. A study found that ozone affects disease progression in patients with sepsis and increases the risk of acute respiratory distress syndrome (13). Furthermore, a United States study showed that exposure to elevated ozone levels is associated with a higher risk of mortality in sepsis patients. Our study extends this finding to a broader population (24). Although ozone concentrations in China are much higher than the global average level (25) and have shown an upward trend (26), only a small number of studies in China have examined the risk of sepsis associated with ozone exposure. One study found a positive association between short-term ozone exposure and sepsis-related hospital admissions (14), while another study showed that this association was even more pronounced in patients with pulmonary sepsis (15). To date, Chinese research has not paid attention to its effect on mortality in sepsis patients. Our study found that ozone exposure increases the risk of mortality in sepsis patients, thereby further extending the evidence on the relationship between ozone exposure and sepsis.

For better air quality management, the World Health Organization has established a series of long-term and short-term exposure guideline values for air pollutants, such as particulate matter, nitrogen dioxide, and sulfur dioxide (27). However, no long-term guideline value has been set for ozone; instead, only short-term limits based on the daily maximum 8 h average and 24 h average have been established. This may be due to insufficient evidence to recommend guideline values for annual mean concentrations. In our study, we found that when the annual average ozone concentration exceeded 89.65 μg/m^3^, the risk of mortality increased significantly. These findings may support the development of annual average ozone concentration standards, highlighting the adverse health effects associated with long-term exposure to high ozone levels. It is crucial to strengthen long-term management of ozone concentrations in the future.

The potential mechanisms by which ozone pollution may contribute to mortality risk in sepsis patients remain to be elucidated. One possible explanation is that this association is mediated by inflammation and oxidative stress. Ozone, as one of the most potent oxidants among air pollutants (16), can cause multi-organ damage by inducing the release of inflammatory factors and increasing the oxidative stress level (18, 28). Furthermore, sepsis patients have an imbalance in their redox state, characterized by excessive production of reactive oxygen species and reactive nitrogen species, mitochondrial dysfunction, and a breakdown of antioxidant systems (29). At present, the pro-inflammatory effects of ozone in the context of sepsis have been observed in animal experiments (30). Further exploration is needed in the future.

The overall mortality in this study cohort was lower than that reported for sepsis hospitalizations in previous literature. For example, a large national study (31) reported in-hospital mortality rates of 4.8% in minors, 17.4% in adults, and 40.8% in older adults. Given the predominance of children in this cohort, who have a significantly lower baseline risk of death from sepsis than older patients, the lower overall mortality observed in this study is probably due to the higher proportion of younger individuals. Recognizing age as a key determinant of sepsis outcomes, we conducted stratified analyses to assess heterogeneity in the ozone–mortality association across age subgroups. We found that children seemed to have a higher risk of mortality among sepsis patients. Previous studies have also reported age differences in the association between ozone exposure and hospitalization or death, indicating that the association is more significant in children (32–34). This difference mainly stems from the unique respiratory physiological characteristics of children. Minute ventilation per body weight is significantly higher in children than in adults (35), despite their relatively low body surface area. At the same time, their developing immune system may have difficulty coping effectively with the oxidative stress and inflammatory response triggered (36). In addition, children are generally more active outdoors, which may further increase the duration of ozone exposure (37, 38).

Beyond age, sex is another key demographic factor that may modify susceptibility. Multiple studies have found that female individuals are more vulnerable to the effects of ozone exposure (39–41). Accumulating evidence indicates significant sex-specific differences in the inflammatory and immune responses to ozone. Epidemiologic observations suggest that female individuals may exhibit heightened sensitivity in specific cytokine pathways (TNFα activation) caused by ozone exposure (42). Although airway hyper-responsiveness (AHR) was less pronounced in female mice than in male mice, they showed more severe lung inflammation, characterized by distinct differences in the expression patterns of neutrophil chemokines, IL-6, and oxidative stress-related enzymes (43, 44). Further mechanistic studies have shown that there are also sex differences in ozone-induced oxidative stress and activation of the hypothalamic–pituitary–adrenal (HPA) axis, which may regulate systemic inflammation and cardiopulmonary outcomes through neuroendocrine feedback (45). These physiological differences, likely rooted in variations in hormone levels and body composition between sexes (46), highlight the importance of targeting specific populations for prevention and control.

Our study has several strengths. First, it is the first to investigate the association between ozone exposure and the risk of mortality in sepsis patients in China. Second, air pollutant data were obtained from the CHAP dataset, and the spatial resolution of the dataset is 1 × 1 km^2^, which means a more accurate assessment of exposure. Finally, the use of multiple adjusted logistic regression models enhanced the robustness of the results, and we also accounted for the impact of PM. However, our study still has several limitations. First, our data on sepsis patients were obtained from medical institutions in Hunan Province and may not be representative of the entire population of mainland China. While our results are consistent with studies conducted in other countries, validation in other regions of China is needed. Second, we included particulate matter in our two‑pollutant model without adjusting for other air pollutants (nitrogen dioxide, sulfur dioxide, and carbon monoxide). Nevertheless, our results remain valid to some extent, because previous studies have suggested that particulate matter has the greatest effect on mortality in sepsis patients (10). Moreover, previous studies have not reported a clear increase in mortality risk associated with these pollutants among sepsis patients. Third, air pollutant data were matched based on self-reported current addresses, which may introduce reporting bias. Further studies with more precise address ascertainment are needed to address this limitation. Fourth, ozone concentrations are strongly influenced by temperature, and their health effects may differ between warm and cold seasons (47), which was not accounted for by the annual average ozone exposure concentration used in this study. Further studies could explore the effects of seasonal ozone exposure on mortality in patients with sepsis. Finally, residual confounding may persist due to the lack of data on diet, lifestyle, genetic predisposition, and indoor air quality, which should be considered when interpreting our findings.

## Conclusion

Long-term ozone exposure is associated with an elevated risk of mortality among sepsis patients, especially among female individuals and children. Our study suggests that ozone is a potentially modifiable risk factor for mortality in this population, providing evidence to inform policy development and management strategies. Future studies at the national level are needed to confirm our findings and explore potential mechanisms.

## Data Availability

The raw data supporting the conclusions of this article will be made available by the authors, without undue reservation.

## Footnotes

1
https://weijing-rs.github.io/product.html
