# Supplementary material for: Long-term ozone exposure and the risk of mortality in sepsis patients in a Chinese cohort
Source: Front Public Health. 2026 Jun 19;14:1782393. doi: 10.3389/fpubh.2026.1782393 (PMC13328280; doi:10.3389/fpubh.2026.1782393)
Supplement: Supplementary file 1 [file Supplementary_file_1.doc]

**Supplementary material**

**Table legend**

**Table S1. Ozone concentration distribution in other years.**

**Table S2. The association between ozone exposure and the level of C-reactive protein in patients with sepsis.**

**Table S3. Associations between particulate matter and the risk of mortality in sepsis patients.**

**Figure legend**

**Figure S1. Flow chart for the selection of patients with sepsis.**

**Figure S2. Associations between the annual average ozone exposure and the risk of mortality in sepsis patients.**

**Figure S3. The association between ozone exposure and severe and complicated conditions in sepsis patients.**

**Figure S4. Associations between ozone exposure and the risk of mortality in sepsis patients without comorbidity.**

**Table S1. Ozone concentration distribution in other years.**

| **O_3_(μg/m^3^)** | **Total population** | **Deaths** | **Survivors** | **P** |
| --- | --- | --- | --- | --- |
| prior 0-years | 91.7(89.8,97.4) | 98.9(92.2,99.9) | 91.7(89.8,96.5) | <0.001 |
| prior 1-years | 89.7(87.2,55.9) | 95.6(91.1,96.7) | 89.7(87.2,94.8) | <0.001 |
| prior 2-years | 89.9(88.2,93.7) | 93.2(92.4,94.5) | 89.1(88.2,93.5) | <0.001 |
| prior 3-years | 87.1(85.5,94.1) | 94.0(92.4,95.7) | 86.9(85.5,94.1) | <0.001 |
| prior 4-years | 84.6(82.7,92.6) | 93.7(91.1,94.0) | 84.4(82.7,92.0) | <0.001 |
| prior 5-years | 84.3(82.6,91.2) | 92.3(89.4,92.6) | 84.3(82.6,90.1) | <0.001 |

Abbreviations: O_3_, ozone.

**Table S2. The association between ozone exposure and the level of C-reactive protein in patients with sepsis.**

| **CRP** | **n** | **β (95% CI)** | **P** |
| --- | --- | --- | --- |
| All population | 2576 | **0.503(0.023,0.984)** | **0.040** |
| Sex group |  |  |  |
| Male | 1565 | 0.331(-0.288,0.950) | 0.294 |
| Female | 1011 | 0.686(-0.084,1.456) | 0.081 |
| Age groups |  |  |  |
| Children | 2007 | **0.547(0.129,0.965)** | **0.010** |
| Adolercents | 44 | 3.198(-1.752,0.965) | 0.198 |
| Adults | 258 | 0.347(-1.874,2.567) | 0.759 |
| Older Adults | 267 | 0.415(-1.518,2.349) | 0.673 |

Adjusted for baseline age, sex, operation, hospital days, pneumonia, upper respiratory tract infection (URTI), diabetes, hypertension, intracranial infection. CRP: C-reactive protein.

**Table S3. Associations between particulate matter and the risk of mortality in sepsis patients.**

| **Adjusted model** | **OR（95%CI)** | **P** |
| --- | --- | --- |
| Prior 1-years PM_1_ | 1.157(0.990,1.351) | 0.067 |
| Prior 1-years PM_2.5_ | 1.085(0.999,1.178) | 0.052 |
| Prior 1-years PM_10_ | 1.070(1.010,1.133) | 0.022 |

Abbreviations: PM_1_, particulate matter with aerodynamic diameter ≤ 1μm; PM_2.5_, particulate matter with aerodynamic diameter ≤ 2.5 μm; PM_10_, particulate matter with aerodynamic diameter≤ 10 μm; OR, odds ratio; CI, confidence interval. Adjusted model: adjusted for baseline age, sex, operation, hospital days, pneumonia, upper respiratory tract infection (URTI), diabetes, hypertension, intracranial infection.

**Figure S1. Flow chart for the selection of patients with sepsis.**


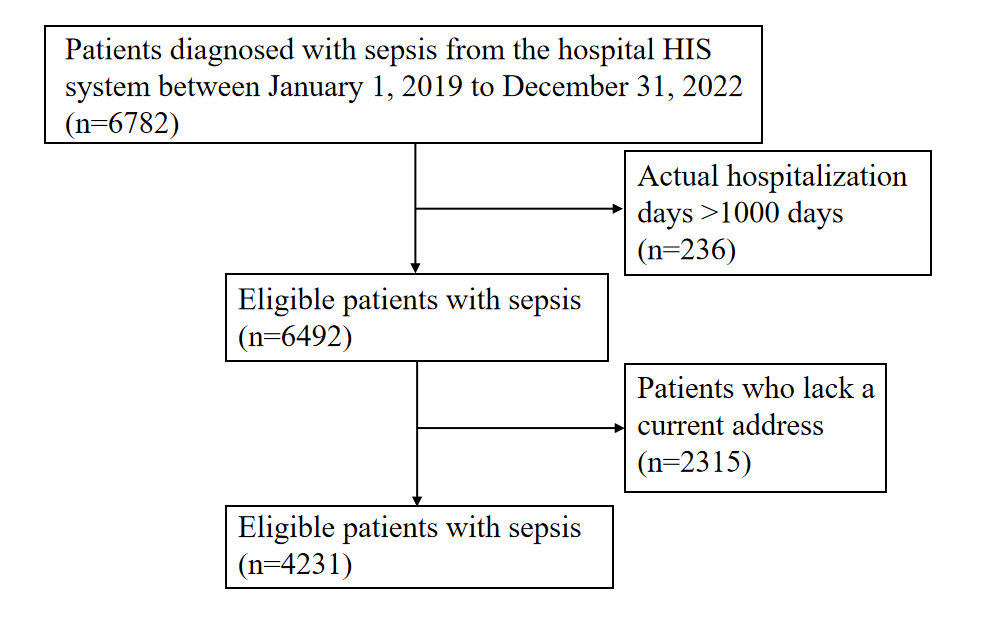


**Figure S2. Associations between the annual average ozone exposure and the risk of mortality in sepsis patients.**


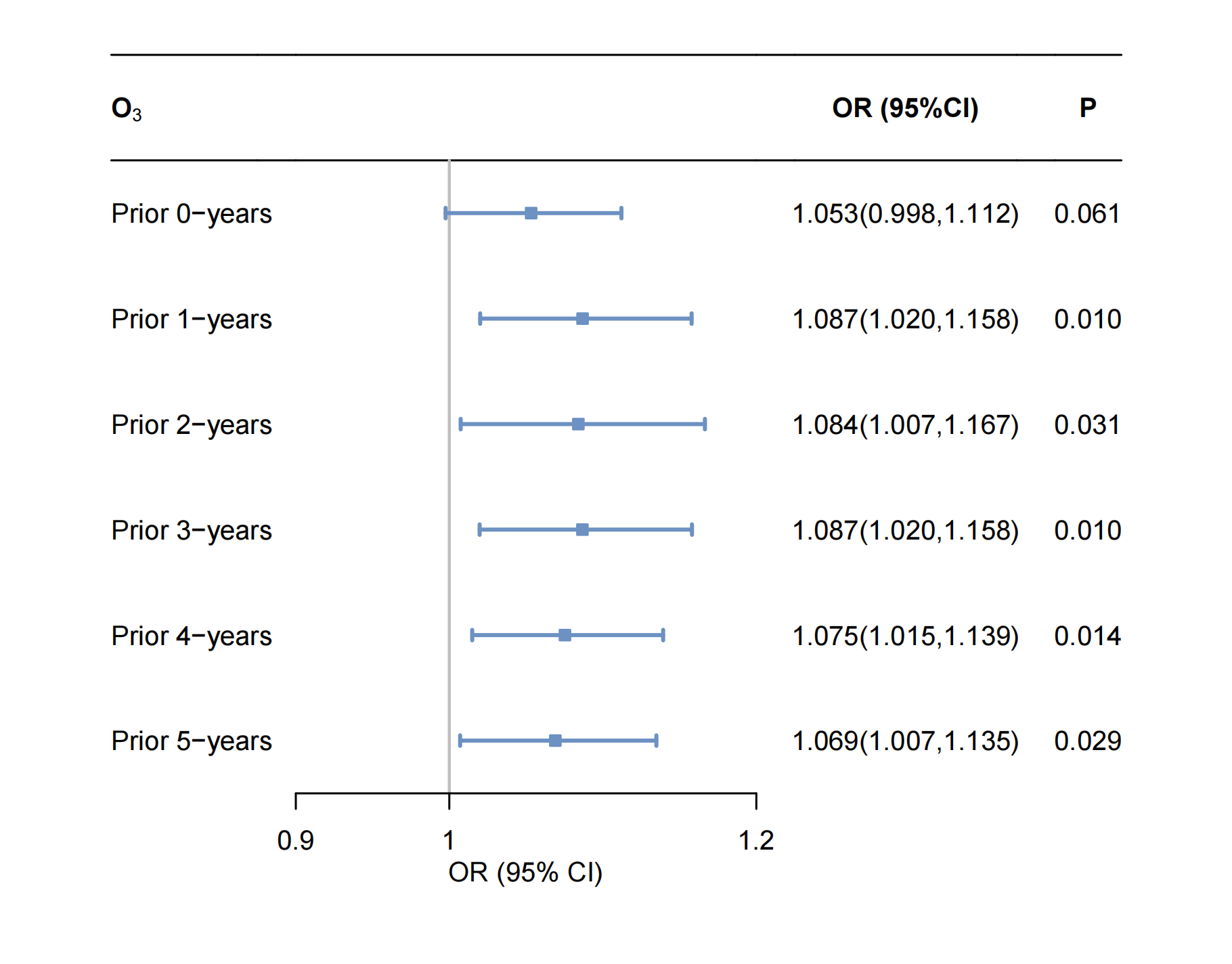


Adjusted model: adjusted for baseline age, sex, operation, hospital days, pneumonia, upper respiratory tract infection (URTI), diabetes, hypertension, intracranial infection;

Abbreviations: O_3_, ozone; OR, odds ratio; CI, confidence interval.

**Figure S3. The association between ozone exposure and severe and complicated conditions in sepsis patients.**

Adjusted model: adjusted for baseline age, sex, operation, hospital days, pneumonia, upper respiratory tract infection (URTI), diabetes, hypertension, intracranial infection; Abbreviations: OR, odds ratio; CI, confidence interval.

**Figure S4. Associations between ozone exposure and the risk of mortality in sepsis patients without comorbidity.**


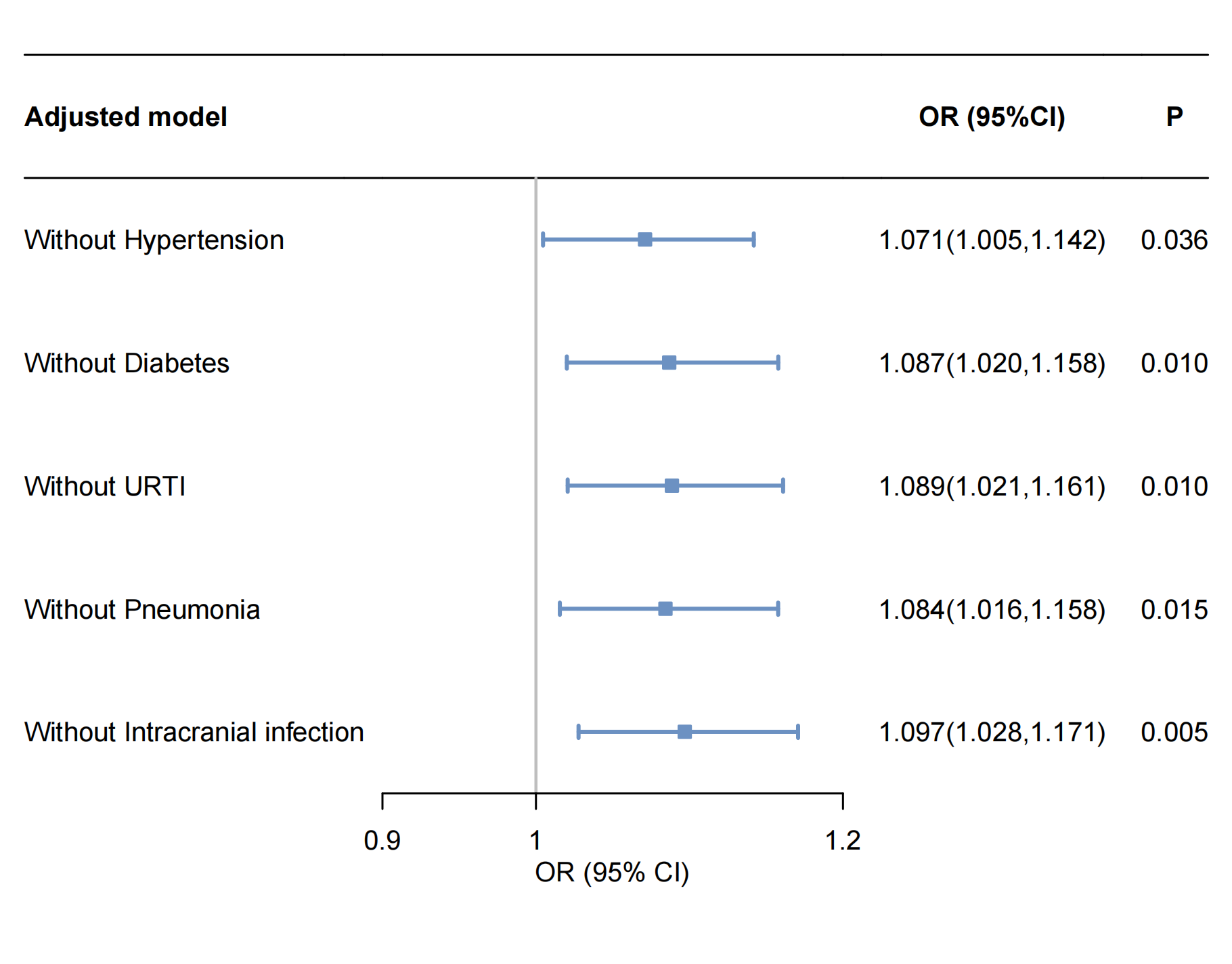
Adjusted model: adjusted for baseline age, sex, operation, hospital days, pneumonia, upper respiratory tract infection (URTI), diabetes, hypertension, intracranial infection. Abbreviations: OR, odds ratio; CI, confidence interval.
